# 589 Reduced Mortality with Use of Point of Care Cell Suspension Autograft

**DOI:** 10.1093/jbcr/iraf019.218

**Published:** 2025-04-01

**Authors:** Muzamil Ahmad, Soman Sen, Kathleen Romanowski, Tina Palmieri, David Greenhalgh, Jason Heard

**Affiliations:** William Beaumont School of Medicine, Oakland University; University of California, Davis and Shriners Hospitals for Children; University of California, Davis and Shriners Hospitals for Children; University of California, Davis and Shriners Hospitals for Children; Shriners Children’s Northern California; University of California Davis

## Abstract

**Introduction:**

Cell suspension autograft (CSA) is a non-cultured, autologous cellular suspension of skin cells that can be used as a primary treatment of partial thickness burns as well as an adjunct to widely meshed split thickness skin grafts (STSG). Literature has demonstrated improved patient outcomes with CSA, contributing to its growing utilization in burn treatment; however, there remains a gap regarding its impact on patient outcomes when used as an adjunct to widely meshed grafts and mortality. The aim of this study is to investigate the clinical efficacy of CSA in relation to mortality, length of stay, and number of surgical procedures. We hypothesize that patients treated with CSA and STSG will have improved outcomes including reduced mortality rates compared to those treated with STSG alone.

**Methods:**

A retrospective, matched, case-control study was conducted in adult burn patients admitted to a regional burn center from 2015 to 2023. A de-identified burn registry was used to create two study groups: patients treated with CSA and STSG (n=63, “CSA-treated”) versus patients treated with STSG alone (n=126, “Controls”). Controls were matched in a 2:1 fashion to CSA-treated patients based on third-degree total body surface area burned (TBSA) and age. Data points extracted from the registry include: TBSA; second-degree TBSA; third-degree TBSA; presence of inhalation injury; number of operations; length of stay (LOS); intensive care unit length of stay (ICU LOS); and mortality. Chi-squared tests were performed for univariate analysis. Multivariate linear regression was used to analyze continuous outcomes. Multiple logistic regression was used to analyze binary outcomes.

**Results:**

189 patients were included for analysis: 63 CSA-treated and 126 controls. There were no significant differences in third-degree TBSA, age, or inhalation injury between the two groups. Multiple logistic regression controlling for third-degree TBSA, age, and inhalation injury, demonstrated a significant reduction in mortality (p=0.0445) and a 78.9% reduction in odds of death (OR: 0.211) for CSA-treated patients compared to controls. CSA-treated patients displayed a non-significant increase in LOS (p=0.0670) and ICU LOS (p=0.0851) over controls. No significant difference was seen in the number of procedures between the two groups (p=0.9084).

**Conclusions:**

The use of CSA in combination with STSG in burn treatment is associated with significant reductions in mortality and odds of death compared to patients treated without CSA. Selection bias may account for the improved mortality in the CSA-treated group and non-significant increases in LOS, ICU LOS, and number of procedures may be reflective of increased survivorship.

**Applicability of Research to Practice:**

CSA and STSG combination treatment may improve survival in burn patients. However, further research is necessary to validate these findings.

**Funding for the Study:**

N/A

